# End stage renal disease in patient with microscopic polyangiitis and atypical hemolytic-uremic syndrome arose 3 weeks after the third dose of anti-SARS-CoV2 vaccine mRNA-1273: A case report with literature revision

**DOI:** 10.1097/MD.0000000000036560

**Published:** 2023-12-15

**Authors:** Veronica Moronti, Francesco Carubbi, Laura Sollima, Luca Piscitani, Claudio Ferri

**Affiliations:** a University of L’Aquila, Department of Life, Health and Environmental Sciences; Internal Medicine and Nephrology Division, ASL 1 Avezzano-Sulmona-L’Aquila, San Salvatore Hospital, L’Aquila, Italy; b Anatomy and Pathological Histology Division, ASL 1 Avezzano-Sulmona-L’Aquila, San Salvatore Hospital, L’Aquila, Italy; c Nephrology and Dialysis Division, Department of Medicine, ASL 1 Avezzano-Sulmona-L’Aquila, San Salvatore Hospital, L’Aquila, Italy.

**Keywords:** aHUS, case report, end-stage renal disease, MPA, SARS-COV2 vaccine

## Abstract

**Rationale:**

Immune system deregulation, including AAV, is a key event that may potentially evolve into ESRD. Abnormal activation of the cAP is also a cardinal feature of TMA, particularly aHUS. The kidney is the most frequently involved organ, and renal-limited forms of TMA are often encountered in clinical practice. Isolated case reports described the occurrence of renal TMA in AAV patients. Some cases of both de novo and relapses of AAV and/or TMAs after anti-SARS-CoV2 vaccination have been reported. We reported, for the 1^st^ time, a case of patients with new-onset MPA and aHUS occurring 3 weeks after the third dose of mRNA-1273 vaccine anti-SARS-CoV2.

**Patient concerns:**

We present a 67-year-old man, affected by arterial hypertension, reported, after mRNA-1273 vaccine anti-SARS-CoV2, anuria, fatigue, anorexia and nausea. Laboratory data revealed acute renal failure.

**Diagnosis:**

Positivity of MPO-ANCA was observed. 7 days after admission, we observed a worsening of anemia and thrombocytopenia with haptoglobin reduction, LDH increase and presence of schistocytes. Plasma levels of ADAMTS-13 were normal. A renal biopsy was performed, and findings were consistent with microscopic polyangiitis, with features of micro-thrombotic glomerulopathy. Genetic tests revealed absence of hybrid genes associated with the increased risk of aHUS.

**Interventions and outcomes:**

We started renal replacement treatment, including hemodialysis, and pulsed methylprednisolone, with no improvement of laboratory parameters. Then, plasma exchange was performed leading to partial haematological response. Only with Eculizumab, a human C5 inhibitor, we observed a normalization of haptoglobin levels and platelets’ count. However, three months after discharge, the patient still required hemodialysis.

**Lessons:**

To our knowledge we observed the first case aHUS, without genetic predisposition, associated with MPA occurring after the third dose of anti-SARS-CoV2 vaccine. This case report highlights the potential link between anti-SARS-CoV2 vaccine as a trigger of MPA and aHUS. This systematic review offers additional perspectives. It is plausible to hypothesize that the vaccine was the trigger for the development of these 2 diseases.

Solid evidence on the mechanisms of interaction between vaccine and immune system, the role of genetic predisposition, and other variables, will shed additional light on the controversial link between anti-SARS-CoV2 vaccine and autoimmunity.

## 1. Introduction

Although in developed countries diabetes mellitus is the leading cause of end-stage renal disease (ESRD), many other conditions may induce kidney damage and culminate into ESRD.^[1]^ Immune system deregulation is a key event shared by several of the conditions that may potentially evolve into ESRD, including the autoantibody-induced glomerular disease caused by antineutrophil cytoplasmic antibody (ANCA)-associated vasculitis (AAV).^[2–5]^

The term AAV encompasses different conditions affecting small vessels (i.e., capillaries, venules, arterioles, and small arteries) and characterized by necrotizing vasculitis, a few, if any, immune complexes and ANCA positivity. AAV include granulomatosis with polyangiitis (GPA), microscopic polyangiitis (MPA), eosinophilic granulomatosis with polyangiitis (EGPA), and renal-limited vasculitis.^[6–8]^ The latter is characterized by isolated crescentic glomerulonephritis and no other organ involvement.^[9]^ With regard to renal involvement, the frequency varies according to the subtype of AAV.^[10–12]^

Historically, it was commonly accepted that serum C3 (sC3) and C4 (sC4) levels are normal in patients with AAV and this probably led to an underestimation of the pathogenic relevance of the complement system in AAV.^[13]^ In fact, studies in humans and experimental models demonstrated an activation of the terminal part of the complement cascade, particularly of C5, with an engagement of the C5a receptor.^[14–16]^ Furthermore, immunohistological studies confirmed the deposition of C3d, factor B, and factor P in glomeruli and small blood vessels.^[17,18]^

Abnormal activation of the complement alternative pathway (cAP) is also a cardinal feature of thrombotic microangiopathy (TMA), particularly atypical hemolytic uremic syndrome (aHUS).^[13]^ TMA is characterized by endothelial cell injury, intravascular platelet-fibrin thrombi, and vascular damage, leading to ischemic organ injury, thrombocytopenia, and microangiopathic hemolytic anemia.^[19]^ The kidney is the most frequently involved organ, and renal-limited forms of TMA are often encountered in clinical practice.^[20]^ Despite difficulties in the classification, TMAs can be divided into primary and secondary forms. In primary forms, a defined abnormality with known pathophysiology is the probable cause and they include thrombotic thrombocytopenic purpura (TTP) (acquired and hereditary) and complement-mediated TMA also known as aHUS, Shiga toxin–mediated hemolytic uremic syndrome, drug-induced TMA and rare hereditary metabolism and coagulation-mediated TMA. It also occurs secondarily as a complication of several systemic conditions, including disseminated intravascular coagulation, cancer, malignant arterial hypertension, infections, hematopoietic stem cell or organ transplantation, autoimmune diseases and pregnancy-associated syndromes (severe preeclampsia/HELLP syndrome).^[19,21–23]^

In particular, aHUS is a chronic, progressive and life-threatening disorder caused by a genetic or acquired disruption in the regulation of the alternative pathway of the complement system.^[24]^ This condition is rare, with a reported incidence of approximately 0.5 per million per year.

Interestingly, isolated case reports described the occurrence of renal TMA in AAV patients, an association that may pose diagnostic and therapeutic challenges.^[25–31]^ In addition, some cases of both de novo and relapses of AAV and/or TMAs after anti-SARS-CoV2 vaccination have been reported^[32–53]^

Here we described for the first time a case of new-onset MPA and aHUS occurring 3 weeks after the third dose of anti-SARS-CoV2 vaccine (mRNA-1273). A literature revision was also performed. Written informed consent was obtained from the patient for publication of this case report and accompanying images.

## 2. Case presentation

A 67-year-old man was referred to our Unit with anuria, fatigue, anorexia and nausea that appeared in the previous 10 days. His past medical history was only characterized by arterial hypertension.

Three weeks before the onset of this clinical pictures, the patient received the third dose of anti-SARS-CoV2 vaccine mRNA-1273. The previous two doses he received were ChAdOx1 (first dose) and mRNA-1273 (second dose).

At physical examination upon hospital admission, the patient showed a body temperature of 36.5°C, arterial blood pressure 160/70 mm Hg, heart rate 72 beats per minute (bpm), respiratory rate 23/min and blood oxygen saturation by pulse oximetry 97% (fraction of inspired oxygen, FiO2, 21%). Chest and abdomen examination did not reveal any abnormalities, but dependent edema was present.

Laboratory data at the admission are summarized in Table 1, showing an acute renal failure therefore we started renal replacement treatment, including hemodialysis, since the first day.

**Table 1:** Laboratory data in the first days after admission.

Glycemia	102 mg/dL
Blood urea nitrogen	409 mg/dL
Creatinine	21.23 mg/dL
Sodium	139 mEq/L
Potassium	5.3 mEq/L
Calcium	7.9 mg/dL
Phosphorous	4.6 mg/dL
Total bilirubin	0.92 mg/dL
Direct bilirubin	0.39 mg/dL
Indirect bilirubin	0.53 mg/dL
Hemoglobin	10.1 g/dL
Mean corpuscolar volume	90.5 fL
Mean corpuscolar hemoglobin concentration	34.2 g/dL
Platelets	132 × 10^3/UL
Blood sedimentation rate	107 mm/h
Ferritin	2182,6 ng/mL
Vitamin B12	386 pg/mL
Folic acid	<2.2 ng/mL
Erythropoietin	12.47 mUI/mL
Parathyroid hormone	187.3 pg/mL
Procalcitonin	1.35 ng/mL
Protein C reactive	9.98 mg/dL
Lactate dehydrogenase	369 UI/L
Albumin	3.23 g/dL
Haptoglobin	<8 mg/dL
Antithrombin III	106%
D-dimer	>4 µg/ml
p-ANCA (MPO)	394.9 UA/mL
c-ANCA (PR3)	0.0 UA/mL
ANA	<1:40
Anti-ENA screening	negative
C3	54 mg/dL
C4	18 mg/dL
C1-inactivator	0,40 g/L
BNP	2979.2 pg/mL

ANA = antinuclear antibodies, ANCA = anti-neutrophil cytoplasmic antibodies, anti-ENA = autoantibodies to extractable nuclear antigens, BNP = brain natriuretic peptide, C = complement, MPO = myeloperoxidase, PR3 = proteinase 3.

In addition, p-ANCA positivity was detected in ethanol-fixed neutrophils by immunofluorescence with antibodies directed against myeloperoxidase (MPO) identified by immunoassay.

A chest computed tomography (CT) showed paraseptal emphysema, mostly present in the upper lobes; no alterations in the density of the parenchyma due to inflammatory and/or infiltrative processes were detected. An abdomen CT described only mild hepatomegaly. An electrocardiogram revealed sinus rhythm with a heart rate of 80 bpm, while transthoracic echocardiogram showed concentric parietal hypertrophy of the left ventricle, ejection fraction of 60%, normal volume of the left atrium, normal caliber of the ascending aorta, no right ventricle dilation, impaired diastolic relaxation; mild mitral and tricuspid regurgitation with systolic pulmonary artery pressure 50 mm Hg.

Seven days after admission, we observed a worsening of anemia and thrombocytopenia with haptoglobin reduction, lactic dehydrogenase increase and presence of schistocytes on morphological examination of peripheral blood. Since the patient did not experience diarrhea, Escherichia coli Shiga toxin was not investigated whereas other microbiological tests for detecting an infectious trigger were negative, and plasma levels of a disintegrin and metalloprotease with thrombospondin type I repeats (ADAMTS)-13 were normal. Taking into account ADAMTS13 normal level and absence of diahrroea, aHUS was a plausible diagnosis.^[54]^

**Figure 1.**
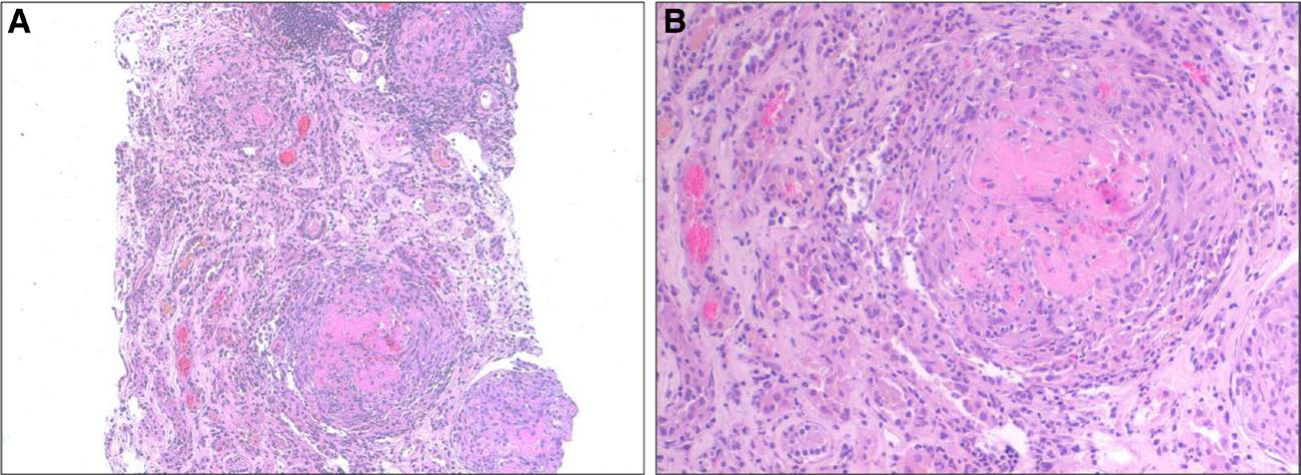
Histological findings in renal biopsy. Intraglomerular thrombi, fibrinoid necrosis with neutrophilic granulocyte infiltrate and fibrocellular crescents; tubular atrophy and presence of intratubular hyaline cylinders. Interstitial mild edema with moderate inflammatory infiltrate in the periglomerular area, consisting of small lymphocytes, neutrophil granulocytes, histiocytes and some plasma cells (A. 10×; B. 20×). H&E = hematoxylin and eosin.

A renal biopsy was performed, with evidence of intraglomerular thrombi, fibrinoid necrosis with neutrophilic granulocyte infiltrate and fibrocellular crescents; tubular atrophy and presence of intratubular hyaline cylinders. Interstitial mild edema was also observed alongside a moderate inflammatory infiltrate in the periglomerular area consisting of small lymphocytes (CD3 +/−, CD20 −/+), neutrophil granulocytes, histiocytes (CD 68+) and some plasma cells. These findings were consistent with microscopic polyangiitis, with features of micro-thrombotic glomerulopathy (Fig. 1).

Genetic tests for aHUS predisposition (Multiplex ligation-dependent probe amplification assay for research deletion/duplication of Complement Factor H (CFH) and CFH-related (CFHR) 1, 2, 3, 4, 5 genes) revealed a polymorphic deletion of CFHR3/CFHR1, frequently mutated in the general population, and absence of hybrid genes associated with the increased risk of aHUS.

Twelve days after admission, due to hypoxemia, the patient required high-flow oxygen therapy and chest CT showed bilateral dependent pleural effusion, areas of reduced ventilation in the perihilar area with some patchy areas of consolidation. These findings were compatible with initial interstitial-alveolar edema with inflammation but the patient never experienced hemoptysis during in-hospital stay.

Taking together all the aforementioned findings, a diagnosis of MPA was made (the patient also fulfilled the 2022 American College of Rheumatology/European Alliance of Associations for Rheumatology classification criteria for microscopic polyangiitis).^[7]^

The patient was treated with pulsed methylprednisolone (1000 mg/d for 3 consecutive days), subsequently reduced to 80 mg/d (1 mg/kg) for 7 days, however no improvement of the renal and haematological parameters was observed. Plasma exchange was performed leading to partial haematological response (schistocytes disappeared), however, anemia, low platelets count and end stage renal disease persisted.

Twenty-one days after hospitalization, due to the worsening of the haematological pictures, we started eculizumab, a human C5 inhibitor, 600 mg/wk the first 2 weeks, then, 900 mg at the 3 week, and after 900 mg every 14 days. A normalization of haptoglobin levels and platelets’ count was observed after 6 weeks of therapy and the patient was eventually discharged.

In the 3 months after discharge laboratory tests documented stabilization of haematological parameters, and also hemoglobin was within the normal range. Furthermore, glucocorticoid therapy was tapered until withdrawal 4 weeks after discharge and p-ANCA were negative. Eculizumab therapy was maintained with no aHUS flare. Unfortunately, however, 3 months after discharge, renal function did not return to normal and the patient still required hemodialysis due to persistent anuria.

## 3. Discussion

He we described for the first time a case of new-onset MPA and aHUS, occurring 3 weeks after the third dose of anti-SARS-CoV2 vaccine.

AAV prevalence is 200 to 400 cases per million people,^[55–58]^ and renal involvement varies according to AAV subtypes, occurring in about 90% of MPA patients, 80% of GPA patients, and 45% of EGPA patients.^[11]^ Interestingly, MPA has a 5-year survival rate poorer than that of EGPA and GPA, likely resulting from renal or lung impairment at disease onset.^[10,59]^

aHUS is a rare condition within the TMA spectrum, with a reported incidence of approximately 0.5 per million per year.^[24]^ HUS, which is characterized by nonimmune hemolytic anemia, thrombocytopenia, and renal impairment, mainly occurs in young children and in most cases, it is secondary to Escherichia coli O157 H7 and other Shiga-toxin–producing strain infection. However, approximately 10% of cases are atypical and not associated with infection.^[60]^ Kidneys are the most frequently injured organs, with acute renal failure, presence of platelet thrombi in the renal microcirculation (microthrombotic glomerulopathy), and renal-limited forms of TMA are not infrequent in clinical practice.^[61]^ ESRD or death occurs in approximately 33% to 40% of patients during the first clinical manifestation of aHUS.^[62–64]^

In our case, coexistence of MPA with high p-ANCA (MPO) concentration (394.9 UA/mL) and aHUS led to renal failure.

MPA and aHUS share similar pathogenic mechanisms such as endothelial dysfunction and cAP dysregulation. In particular, endothelial cells are an important target of both AAV and TMAs, contributing to the development of renal damage.^[65,66]^ Endothelial injury results in a prothrombotic state through exposure of subendothelial collagen, von Willebrand factor and fibrinogen. Moreover, a previously unknown role for complement, particularly C5a in ANCA-induced neutrophil activation has been recently suggested.^[16,67–70]^ Interestingly, abnormal cAP activation is also a cardinal feature of TMA, and the hallmark of the aHUS.^[13]^ These defects are either inherited, acquired, or a combination of the two, and they result in chronic, uncontrolled activation of the complement system.^[60,71–73]^ This leads to platelet, leukocyte, and endothelial-cell activation and ultimately to systemic thrombotic microangiopathy.^[60,62,63,74–76]^

Genetic analyses in the CFH/CFHRs aHUS predisposition revealed that our patient had no predisposition to aHUS, since the polymorphic deletion of CFHR3/CFHR1 is frequently observed in general population.

TMA, especially aHUS, in patients with AAV has been mainly reported in isolated case reports and retrospective studies (Table 2).

**Table 2:** Reported cases of patients with AAV and aHUS.

Reference	Patients	AAV diagnosis	aHUS diagnosis
Stefanidis I, 1998^[27]^	Female patient, 68 years	PR3-ANCA- Remittent intra- and extracapillary RPGN followed by the characteristic lesions of HUS	Laboratory findings and histological findings
Chen SF, 2015^[77]^	30 patients (15 males, 15 females) Age ± SD (63.9 ± 11.3)	MPA/GPA/RLV (24/4/2) MPO-ANCA/PR3-ANCA (28/2)	Laboratory findings and histological findings
Sathe KP et Mehta KP, 2016^[78]^	Male patient, 10 years	p-ANCA positive with focal proliferative pauci-immune GN	Laboratory findings
Cavero T, 2017^[79]^	2 Patients Male, 52 years Male, 49 years	EGPA EGPA	Histological findings
Badiola J, 2019^[80]^	Male patient, 57 years	MPO-ANCA positive EGPA. At the skin biopsy: vasculitis with an eosinophilic perivascular inflammatory infiltration without granulomas	Laboratory findings and histological findings
Duong K, 2020^[81]^	Female patient, 82 years	MPO-ANCA positive MPA with early glomerular capillary thrombosis, and cellular crescent at the kidney biopsy	Laboratory findings and histological findings

AAV = antineutrophil cytoplasmic antibodies-associated vasculitis, aHUS = atypical-hemolytic uremic syndrome, ANCA = antineutrophil cytoplasmic antibodies, EGPA = eosinophilic granulomatosis with polyangiitis, GN = glomerulonephritis, GPA = granulomatosis with polyangiitis, HUS = hemolytic uremic syndrome, MPA = microscopic polyangiitis, MPO = myeloperoxidase, PR3 = proteinase 3, RLV = renal-limited vasculitis, RPGN = rapidly progressive glomerulonephritis, SD = standard deviation.

Interestingly, in our patient the initial symptoms started three weeks after the third dose of anti-SARS-CoV-2 vaccine. Associations between anti-SARS-CoV2 vaccines and immune-mediated inflammatory diseases have been reported.^[82]^ With regard to kidney involvement, minimal change disease and IgA nephropathy are the most frequently reported glomerular disease related to anti-SARS-CoV2 vaccination.^[83–87]^ Table 3 shows renal-associated AAV case reports developed after anti-SARS-CoV2 vaccination. However, the causal relationship has not been proven although the post-vaccination onset seems to suggest it.

**Table 3:** Reported cases of renal AAV induced by anti-SARS-CoV2 vaccination.

Article	Age years/sex	COVID-19 vaccine	Onset of symptoms	AAV diagnosis
Anderegg MA, 2021^[32]^	81/Male	mRNA-1273 2nd dose	ND	PR3-ANCA pauci-immune crescentic GN with capillary necrosis and vasculitis present in the renal vessel walls
Dube GK, 2021^[33]^	29/Female	BNT162b2mRNA 2nd dose	16 d	MPO-ANCA pauci-immune crescentic GN
Feghali EJ, 2021^[34]^	58/Male	mRNA-1273 2nd dose	4 d	anti-PR3-associated ANCA GN
Hakroush S, 2021^[35]^	79/Female	BNT162b2mRNA 2nd dose	2 wk	MPO-ANCA associated vasculitis presenting with pauci-immune crescentic GN
Ritter A, 2021^[36]^	69/Male	BNT162b2mRNA 2nd dose	33 d	MPO-ANCA AAV with massive rhabdomyolysis and pauci-immune crescentic GN
Sekar A, 2021^[37]^	52/Male	mRNA-1273 2nd dose	2 wk	PR3-ANCA pauci-immune necrotizing and crescentic GN
Shakoor MT, 2021^[38]^	78/Female	BNT162b2mRNA 2nd dose	2 wk	Renal-limited MPO-AAV
Villa M, 2021^[39]^	63/Male	ChAdOx1 nCoV-19 1st dose	1 wk	P-ANCA-associated pauci-immune GN
Al-Yafeai Z, 2022^[40]^	62/Female	BNT162b2mRNA 1st dose	4 wk	PR3-ANCA-associated vasculitis
Cano-Gámez T, 2022^[41]^	51/Female	ChAdOx1 nCoV-19 3rd dose	ND	MPO-ANCA-associated rapidly progressive GN
Christodoulou M, 2022^[42]^	72/Female	mRNA-1273 2nd dose	2 wk	MPO-ANCA pulmonary–renal syndrome (alveolar hemorrhage and rapidly progressive GN)
Prabhahar A, 2022^[43]^	51/Male	ChAdOx1 nCoV-19 1st dose	15 d	PR-3 AAV with pauci-immune crescentic GN
So D, 2022^[44]^	42/Male	BNT162b2mRNA 2nd dose	ND	MPA with elevated MPO-antibodies and pauci-immune GN
Suzuki M, 2022^[45]^	72/Male	BNT162b2mRNA 2nd dose	1 d	MPO-AAV with severe pauci-immune crescentic GN
Kawamura T, 2023^[46]^	71/Female	BNT162b2mRNA 2nd dose	1 wk	MPO-AAV with manifestations of renal involvement, general symptoms, and the definite presence of MPO-ANCA, as an underlying disease of rapidly progressive GN

AAV = antineutrophil cytoplasmic antibodies-associated vasculitis, AKI = acute kidney injury, ANCA = antineutrophil cytoplasmic antibodies, GN = glomerulonephritis, MPA = microscopic polyangiitis, MPO = myeloperoxidase, PR3 = proteinase 3, RLV = renal limited vasculitis.

Cases of aHUS induced by anti-SARS-CoV2 vaccinations are reported in Table 4.

**Table 4:** Reported cases of aHUS induced by anti-SARS-CoV2 vaccination.

Article	Age/sex	Type of vaccination	Onset symptoms	aHUS diagnosis
Bouwmeester RN, 2022^[47]^	5 Patients 21/Female 58/Female 10/Male 57/Female 53/Male	ChAdOx1 nCoV-19 1st dose ChAdOx1 nCoV-19 2nd dose BNT162b2mRNA 2nd dose BNT162b2mRNA 1st + 3rd BNT162b2mRNA 2nd dose	2 d 3 d 1 d 2 d after first, 10 d after third 40 d	Laboratory findings
Rysava R, 2022^[48]^	21/Female	BNT162b2mRNA 2nd dose	1 d	Laboratory findings
Claes KJ, 2023^[49]^	38/Female	mRNA-1273 booster dose (first and second dose: BNT162b2mRNA)	1 d	Laboratory and histological findings
Tawhari M, 2023^[50]^	38/Male	ChAdOx1 nCoV-19 1st dose	1 wk	Laboratory findings

aHUS = atypical-hemolytic uremic syndrome.

Even though this study presents information about a rare manifestation after anti-SARS-CoV2 vaccination, several limitations exist. First, this was a case report; therefore, generalizations cannot be made. In addition, the number of cases reported in the literature is limited, making it challenging to draw significant epidemiological conclusions. Furthermore, this study could not comment on the possible association between anti-SARS-CoV2 vaccination and MPA and aHUS.

## 4. Conclusion

In conclusion, we observed the first case aHUS, without genetic predisposition associated with MPA occurring after the third dose of anti-SARS-CoV2 vaccine.

Although it is not possible to prove a causal relationship between vaccination and the diseases, it is plausible to hypothesize that the vaccine was the trigger for the development of these two diseases.

Solid evidence on the mechanisms of interaction between vaccine and immune system, the role of genetic predisposition, and other variables, will shed additional light on the controversial link between anti-SARS-CoV2 vaccine and autoimmunity.

## Author contributions

**Resources:** Veronica Moronti.

**Supervision:** Francesco Carubbi, Laura Sollima, Luca Piscitani, Claudio Ferri.

**Writing – original draft:** Veronica Moronti, Francesco Carubbi.

**Writing – review & editing:** Veronica Moronti, Francesco Carubbi.
